# Mitochondrial Dysfunction and Oxidative Stress in Hereditary Ectopic Calcification Diseases

**DOI:** 10.3390/ijms232315288

**Published:** 2022-12-04

**Authors:** Lukas L. Nollet, Olivier M. Vanakker

**Affiliations:** 1 Center for Medical Genetics, Ghent University Hospital, 9000 Ghent, Belgium; 2 Department of Biomolecular Medicine, Ghent University, 9000 Ghent, Belgium; 3 Ectopic Mineralization Research Group Ghent, 9000 Ghent, Belgium

**Keywords:** ectopic calcification, rare diseases, mitochondria, oxidative stress, pseudoxanthoma elasticum, Hutchinson–Gilford progeria syndrome, central nervous system calcification

## Abstract

Ectopic calcification (EC) is characterized by an abnormal deposition of calcium phosphate crystals in soft tissues such as blood vessels, skin, and brain parenchyma. EC contributes to significant morbidity and mortality and is considered a major health problem for which no effective treatments currently exist. In recent years, growing emphasis has been placed on the role of mitochondrial dysfunction and oxidative stress in the pathogenesis of EC. Impaired mitochondrial respiration and increased levels of reactive oxygen species can be directly linked to key molecular pathways involved in EC such as adenosine triphosphate homeostasis, DNA damage signaling, and apoptosis. While EC is mainly encountered in common diseases such as diabetes mellitus and chronic kidney disease, studies in rare hereditary EC disorders such as pseudoxanthoma elasticum or Hutchinson–Gilford progeria syndrome have been instrumental in identifying the precise etiopathogenetic mechanisms leading to EC. In this narrative review, we describe the current state of the art regarding the role of mitochondrial dysfunction and oxidative stress in hereditary EC diseases. In-depth knowledge of aberrant mitochondrial metabolism and its local and systemic consequences will benefit the research into novel therapies for both rare and common EC disorders.

## 1. A Brief Introduction into Ectopic Calcification

While physiological calcification—as is observed in bone and teeth—is of paramount importance in maintaining the structural integrity of the human body, aberrant ectopic calcification (EC) in soft tissues such as arterial blood vessels, skin, or eyes is a pathologic process resulting in significant morbidity and mortality [1]. EC occurs when the balance between systemic and/or local pro-calcifying and anti-calcifying factors tips towards the former, causing a progressive deposition of calcium phosphate crystals—largely hydroxyapatite (Ca_10_(PO_4_)_6_(OH)_2_)—in the extracellular matrix [2].

As calcium and phosphate ion levels in human blood largely exceed their solubility limits, circulating calcification inhibitors such as fetuin-A and inorganic pyrophosphate (PPi) are needed to prevent spontaneous and uncontrolled crystal precipitation [3,4]. Consequently, disease states associated with either hypercalcemia or hyperphosphatemia (as is the case in end-stage renal disease), or with systemic PPi deficiency (as is seen in rare genetic calcification disorders) typically present an EC phenotype [5].

At the local level—that is, in peripheral soft tissues—the formation of calcium crystals is further controlled by the actions of residing mesenchymal cells such as fibroblasts or vascular smooth muscle cells [6]. These cells are actively involved in either preventing or initiating and sustaining the calcification process by enabling local production of anti-calcifying mediators such as PPi and adenosine or by undergoing phenotypic switching towards a calcification-prone cellular state, a process known as osteochondrogenic trans-differentiation [7]. Cells then secrete large amounts of matrix metalloproteinases (MMPs) which enzymatically break down ECM components such as elastic fibers, facilitating crystal deposition on the elastin debris, increase the protrusion of calcium-rich extracellular vesicles from their plasma membrane, and activate pro-osteogenic intracellular signaling pathways including the Bone Morphogenetic Protein (BMP)/Wingless Integrated (Wnt) and Runt-related transcription factor 2 (RUNX2)/Alkaline Phosphatase (ALPL) cascades [8,9].

Clinically, EC results in local tissue damage and subsequent organ dysfunction. For example, medial vascular calcification causes increased arterial stiffness and increased pulse pressure, contributing significantly to left ventricular hypertrophy and heart failure [10]. Calcium crystal deposition in the central nervous system, e.g., in the thalamic nuclei, results in cognitive and behavioral problems that severely affect a patient’s daily functioning and quality of life [1].

While EC may occur in various soft tissues and organs, vascular calcification is by far the most prevalent form of EC and has been studied extensively both in vitro and in vivo [11]. Indeed, many pathophysiological mechanisms of EC were initially identified in studies investigating the pathogenesis of vascular calcification.

In these studies, growing emphasis has recently been placed on the role of mitochondrial dysfunction and oxidative stress in the vascular calcification process [12]. In chronic kidney disease (CKD)-associated calcification mitochondrial metabolic alterations, mitochondrial DNA (mtDNA) damage, and increased levels of reactive oxygen species (ROS) have been reported [13]. Additionally, mitochondrial fusion proteins such as mitofusin-2 play a crucial role in the initiation of VSMC apoptosis by decreasing B-cell lymphoma 2 (Bcl-2) expression and by activating caspase-3 and caspase-9, which are then followed by calcium crystal deposition on the nidus formed by the apoptotic bodies [14]. In calcific aortic valve disease, increased hydrogen peroxide (H_2_O_2_) levels—an end product of ROS metabolism—are associated with accelerated disease progression and increased expression of osteogenic markers such as RUNX2 [15,16,17].

The research fields of vascular calcification (as seen in common acquired diseases) and rare Mendelian EC disorders have long been strongly interconnected. Indeed, studies in hereditary EC diseases have been instrumental in identifying and understanding etiopathogenetic mechanisms leading to EC (e.g., the role of inorganic pyrophosphate (PPi) as a potent endogenous anti-calcifying factor became first apparent in the orphan disease Generalized Arterial Calcification of Infancy (GACI; OMIM #208000)). These mechanisms were also proven to be applicable to the EC process in common acquired diseases and vice versa [18]. Therefore, in-depth knowledge of aberrant mitochondrial and ROS metabolism in the pathogenesis of Mendelian EC diseases is of utmost importance to increase our understanding of common EC diseases and will greatly benefit translational research into novel therapies for both these rare and common EC disorders.

In this narrative review, we describe the current state of the art regarding the role of mitochondrial dysfunction and oxidative stress in hereditary EC diseases (Table 1). Additionally, we formulate multiple outstanding questions which will need to be addressed in future experimental studies.

## 2. Mitochondrial Dysfunction and Oxidative Stress in Pseudoxanthoma Elasticum (PXE)

Of all Mendelian EC disorders, pseudoxanthoma elasticum (PXE; OMIM #264800) is the most common, with an estimated population frequency between 1:25,000 and 1:100,000 [19]. PXE is caused by bi-allelic pathogenic variants in the *ABCC6* (ATP-binding cassette, subfamily C, member 6) gene, which encodes a transmembrane protein responsible for the efflux of a currently unknown substrate [20,21]. The ABCC6 transporter is mainly expressed in non-calcifying cells such as hepatocytes and renal tubule cells, while its expression is considered negligible in calcifying cells such as fibroblasts and VSMCs [22]. ABCC6 mediates the efflux of ATP into the systemic circulation, which is then quickly catalyzed into the calcification inhibitors PPi (by the enzyme ENPP1) and adenosine (converted from AMP by the NT5E protein). Therefore, PXE is considered a metabolic disease due to the systemic deficiency of an elusive anti-calcifying factor, though peripheral cells also contribute actively to the EC process and retain their distinct osteochondrogenic molecular signature even when cultured ex vivo [23,24].

The clinical phenotype of PXE consists of the progressive calcification of elastic fibers throughout the body, resulting in yellowish cutaneous lesions and excessive skin folds, while mineralization of the Bruch’s membrane of the eye causes small breaks called ‘angioid streaks,’ leading to subsequent choroidal neovascularization, bleeding, and vision loss [25]. In the vasculature, medial arterial calcification results in blood vessel occlusion and therefore intermittent claudication [26]. Finally, an increased risk of ischemic stroke and nephrolithiasis is also associated with PXE [3,27].

The involvement of mitochondria in PXE was already hinted at in the early 1990s when microscopy of fibroblasts revealed the destruction of mitochondria in PXE patients [28]. In 2012, it was suggested by Martin et al. that the ABCC6 protein resides in the mitochondria-associated membranes (MAM)—a linkage region between the mitochondrial outer membrane and the endoplasmic reticulum—as opposed to previous studies, which had localized ABCC6 to the basolateral plasma membrane (with mutations in ABCC6 causing aberrant cellular localization, i.e., not in the plasma membrane) [29,30,31]. However, these findings were quickly refuted by Pomozi et al. and Ferré et al., re-establishing the plasma membrane localization of ABCC6, and it was assumed that the use of multi-step fractionation techniques, amongst others, contributed to the erroneous MAM localization [32,33]. On the other hand, the study by Martin et al. revealed a convincing enrichment of mitochondrial gene expression signatures in *Abcc6−/−* mice together with morphological abnormalities in kidney and heart mitochondria (i.e., swollen mitochondria and disrupted and reduced cristae) [29]. Additionally, mitochondrial respiration in liver, kidney, and heart cells from *Abcc6−/−* animals was shown to be severely dysfunctional, evidenced by a significantly reduced oxygen consumption rate following administration of the uncoupler FCCP [29].

These results were later confirmed in PXE patient-derived fibroblasts, in which mitochondria were characterized by partial loss of cristae and concentric ‘onion-like’ cristae [34]. Using live-cell imaging with the MitoView Green stain, the authors further showed that PXE mitochondria formed a more dense network compared to control mitochondria with a significant increase in mitochondrial branching, branch length, and branch junctions, indicative of an unbalance between fission and fusion events, favoring the latter in PXE [34].

The comprehensive study by Lofaro et al. further revealed a reduced oxygen consumption rate in PXE mitochondria similar to Martin et al., characterized by both a lower maximal respiratory rate as well as a decreased spare capacity [34]. The mitochondrial membrane potential ∆ψ_m_, evaluated using ratiometric fluorescence with the JC-1 dye, was shown to be significantly increased in PXE cells compared to controls while mitochondrial proteomics revealed significant changes in expression of proteins involved in redox homeostasis (e.g., SODM and TRAP1), lipid metabolism (e.g., acyl-CoA dehydrogenase), and calcium regulation (e.g., STML2 and MCU) [34]. Strikingly, the inorganic pyrophosphatase 2 (IPYR2) enzyme—which breaks down PPi—was significantly upregulated in PXE mitochondria, creating a potential link between mitochondrial dysfunction and (local) PPi deficiency in PXE [34].

Co-occurring with mitochondrial dysfunction, a mild but chronic oxidative stress is present in PXE both in vitro and in vivo. The Modena group was the first to demonstrate increased intracellular ROS levels in PXE fibroblasts and their attenuation following treatment with the antioxidant vitamin E [35]. Furthermore, malondialdehyde (MDA)—an end-product of lipid peroxidation—was shown to be increased in PXE, as were enzyme activity levels of the mitochondrial manganese superoxide dismutase (MnSOD) [35,36]. The ratio of oxidized to reduced glutathione (GSSG/GSH) was also significantly increased in PXE cells, as was overall protein oxidation status [35]. In PXE mitochondria, increased O_2_^−^ levels, determined using MitoSox-based flow cytometry, were observed compared to controls [34].

At the systemic level, increased oxidative stress remains evident in PXE, demonstrated by higher concentrations of the lipid peroxidation derivative LOOH, advanced oxidation protein products (AOPP), and extracellular SOD activity in blood samples of PXE patients compared to healthy controls [37]. Analogous to the human situation, *Abcc6−/−* mice display increased MDA levels in the liver, decreased total antioxidant status (TAS), and increased protein oxidation in both the liver and serum [38]. As increased oxidative stress is hypothesized to contribute to the EC phenotype in PXE, *Abcc6−/−* mice were treated with an antioxidant diet containing vitamins E and C, selenium, and N-acetylcysteine for 5 months [38]. While this diet significantly reduced oxidative stress markers in the treated animals, no significant decrease in vibrissae calcification—the standard read-out of EC burden in *Abcc6−/−* mice—was observed [38].

ROS are known to cause irreversible and detrimental changes to proteins, lipids, and DNA, thereby affecting the biological function and downstream pathways of these macromolecules. In this context, our group and others have recently demonstrated the involvement of excessive DNA damage response (DDR) signaling, mediated via the PARP1-STAT-IL6-RUNX2 axis, in PXE pathogenesis [39,40]. Indeed, overaction of DDR pathways including ATM, p21, and p53 results in premature senescence in PXE fibroblasts and contributes to the EC process [39,41]. On the contrary, PARP1 inhibition by means of minocycline, which is also a known ROS scavenging molecule, significantly reduced EC in both PXE fibroblasts, zebrafish, and mice models [39,42]. We may therefore hypothesize that increased and chronic oxidative stress in PXE causes persistent low-level DNA damage, activating DDR pathways and accelerating cellular senescence.

Finally, as PXE disease severity is characterized by large intra- and inter-familial variability which cannot be explained by differences in the *ABCC6* genotype itself, modifier genes are believed to substantially influence phenotypic severity. In this regard, an older study from Zarbock et al. showed that common single-nucleotide polymorphisms in the antioxidant genes *SOD2*, glutathione peroxidase 1 (*GPX1*), and catalase (*CAT*) were significantly associated with a younger age of onset of PXE disease, thus highlighting the potential link between redox homeostasis and the development of PXE-related clinical signs and symptoms [43].

### Conclusion and Outstanding Questions

Although the research field of mitochondrial dysfunction and oxidative stress in PXE is still in its infancy, currently available literature supports the hypothesis that the mitochondrial metabolism is pathologically altered in PXE and is accompanied by increased ROS production. However, it remains difficult to evaluate whether mitochondrial dysfunction is either the cause or consequence of EC as increased calcium uptake originating from hydroxyapatite crystals in the ECM may in itself also induce mitochondrial metabolic abnormalities. As impaired mitochondrial respiration has been observed in both liver and kidney cells—which normally express ABCC6—as well as in cells with only minimal ABCC6 expression such as fibroblasts, one may argue that the systemic deficiency of the currently unknown substrate(s) of ABCC6 causes mitochondrial dysfunction remote from the site of its initial production (i.e., the liver and kidneys). This may then initiate increased mitofusin-2 expression resulting in decreased Bcl-2 and enhanced caspase 3-mediated apoptosis. Indeed, we previously showed reduced Bcl-2 and increased caspase-3 expression in PXE fibroblasts [8]. Additionally, mitofusin-2 may accelerate mitochondrial fusion events resulting in increased network formation, explaining the findings of Lofaro et al. in PXE cells. ROS are also known to stimulate the BMP2-SMAD-RUNX2 pathway, a signaling cascade which is highly activated in PXE, as demonstrated earlier by our group [8].

While PXE is considered a PPi-deficiency syndrome due to decreased ABCC6-mediated efflux of ATP into the systemic circulation, resulting in decreased catalysis to PPi by the ENPP1 enzyme, future studies should investigate the contribution of decreased mitochondrial oxidative phosphorylation and therefore reduced ATP production to the PPi deficit. As such, novel combination therapies targeting both decreased ATP production and enhanced ATP breakdown may be developed.

Additional outstanding questions include (I) the presence and role of mtDNA damage in PXE pathogenesis, keeping in mind that mtDNA damage is associated with premature senescence, a hallmark of PXE [44,45]; (II) whether mitochondrial dysfunction progressively worsens with advancing EC; and (III) whether targeting mitochondrial dysfunction and/or oxidative stress ameliorates the EC phenotype and associated clinical signs and symptoms in PXE patients.

## 3. Mitochondrial Dysfunction and Oxidative Stress in β-Thalassemia-Associated EC

The β-thalassemias (OMIM #613985) are a group of inborn blood disorders characterized by reduced or absent beta hemoglobin chains due to mutations in the *HBB* gene. Decreased levels of adult hemoglobin HbA result in microcytic anemia, while splenomegaly and bone malformations may also occur. Treatment typically consists of repetitive blood transfusions which, in turn, cause systemic iron overload. Remarkably, a subgroup of β-thalassemia patients (estimated at around 10–20%) develop a PXE-mimicking phenotype as evidenced by EC in the skin, eyes, and arteries resulting from calcium crystal deposition on elastic fibers [46,47,48].

The underlying molecular etiology causing this striking phenotypic resemblance between these two apparently unrelated Mendelian disorders was further investigated in dermal fibroblasts [49]. Similar to PXE, markers of oxidative stress including superoxide, LOOH, AOPP, and MDA were significantly higher in PXE-like β-thalassemia fibroblasts compared to non-PXE β-thalassemia cells [49]. The same was true for Cu-Zn-SOD and GPX enzyme activity levels [49]. Additionally, undercarboxylated matrix gla protein (ucMGP)—a well-established pro-calcifying factor—was increased in PXE-like β-thalassemia fibroblasts, while concentrations of the anti-calcifying carboxylated MGP (cMGP) were higher in non-PXE β-thalassemia fibroblasts [49]. At the ultrastructural level, ucMGP co-localized with the dense calcified deposits on elastic fibers in skin biopsies from PXE-like β-thalassemia patients, whereas cMGP was found at the interface between calcified and normal elastin, forming a ‘defensive boundary’ against calcium crystals [49].

Intriguingly, while pathogenic variants in the *ABCC6* gene are absent in PXE-like β-thalassemia patients, a mouse model of the disease (*Hbb^th3/+^*) showed a liver-specific downregulation of Abcc6 expression, mimicking the genetic ABCC6-deficiency of PXE [50]. Reduced Abcc6 protein levels in the liver of *Hbb^th3/+^* mice further worsened with advancing age of the animals, suggesting a potential role of Abcc6 in ageing-related EC [50]. Using chromatin precipitation immunoassays, the authors further showed that the association levels of the transcription factor NF-E2 with the *Abcc6* promoter were significantly decreased in *Hbb^th3/+^* livers, which may explain the reduced *Abcc6* transcription in β-thalassemia mice [50,51]. NF-E2 is involved in the regulation of iron homeostasis and globin protein biosynthesis, processes which are both severely disturbed in β-thalassemia [52]. Interestingly, the human orthologue of NF-E2 is also known to be a transcription factor of human *ABCC6*, suggesting that similar transcriptional regulatory mechanisms are also at play in human patients [53].

Finally, none of the *Hbb^th3/+^* mice displayed an EC phenotype, indicating that the relative Abcc6 deficiency is insufficient to induce EC in mice, which are notoriously resistant to soft tissue calcification [50].

Patient-derived fibroblasts from β-thalassemia or other Mendelian EC disease patients maintain their pro-calcifying properties and osteogenic molecular signatures in vitro, even when cultured for multiple passages (>5) [8]. It is widely believed that this phenomenon is either caused by epigenetic alterations obtained in vivo or by rare sequence variants in non-causal genes creating a favorable pro-calcifying genetic background. To further investigate the latter hypothesis, whole-exome sequencing of DNA samples from PXE-like β-thalassemia patients was performed by Boraldi et al., revealing rare sequence variants in genes involved in elastin homeostasis (*FBN3*, *LTBP3*) and mitochondrial functioning (*GPX1*, *SLC25A5*) [54]. As mentioned above, SNPs in *GPX1* have been associated with PXE disease severity, while *SLC25A5* encodes a mitochondrial carrier protein regulating the transport of ATP and ADP between the cytosol and the mitochondrial matrix as well as stabilizing the mitochondrial membrane potential [43,55]. As both aberrant nucleoside triphosphate metabolism and mitochondrial metabolic alterations are hallmarks of EC disorders such as PXE, it is very likely that rare sequence variants in these genes can be linked to the development of a PXE-like phenotype in β-thalassemia patients.

### Conclusion and Outstanding Questions

Soft tissue calcification as observed in β-thalassemia patients presents an interesting model to study the pathogenesis of ‘acquired’ EC, i.e., where no mutations in Mendelian EC-related genes such as *ABCC6* are present. In this context, currently available literature suggests that mitochondrial dysfunction and oxidative stress play a major role in this calcification process. Indeed, it is well-known that β-thalassemia patients suffer from persistent oxidative stress due to the continuous degradation of unstable hemoglobin and systemic iron overload [56]. In turn, the excessive production of free radicals results in accelerated hemolysis and ineffective erythropoiesis, creating a vicious cycle [56]. When compiling the current evidence, we may hypothesize that increased oxidative stress is a common link—and thus potential treatment target—between acquired and hereditary forms of EC.

Apart from β-thalassemia, PXE-like soft tissue calcification (e.g., angioid streaks) has also been reported—although only sporadically—in sickle cell anemia (OMIM #603903), a hemoglobinopathy typically caused by the bi-allelic c.20A > T gain-of-function mutation in the *HBB* gene [57,58]. Interestingly, a phase 3 clinical trial with the antioxidant I-glutamine showed a significantly reduced number of sickle cell-related pain crises in the treatment group compared to placebo, suggesting that oxidative stress in the hemoglobinopathies is amenable to pharmacological therapy with antioxidant compounds [59]. However, whether such treatments also have a beneficial effect on the EC phenotype has yet to be investigated.

## 4. Mitochondrial Dysfunction and Oxidative Stress in Hutchinson–Gilford Progeria Syndrome (HGPS)

Hutchinson–Gilford progeria syndrome (HGPS; OMIM #176670) is an extremely rare (estimated prevalence: 1/20,000,000) autosomal dominant disorder caused by mutations in the *LMNA* (lamin A) gene resulting in an aberrantly spliced protein product termed ‘progerin’ [60]. Considered a paradigm premature ageing disease, HGPS patients quickly develop vascular calcification, osteoporosis, sclerodermatous skin changes, muscle atrophy, and alopecia [61]. As the limited life expectancy—only 13 years—is mainly governed by cardiovascular mortality, current research efforts mainly focus on attenuating the vascular calcification phenotype [62].

HGPS is characterized by systemic PPi deficiency resulting from decreased ATP production and increased PPi hydrolysis via ALPL [63,64]. Rivera-Torres et al. were the first to show that reduced ATP production in HGPS is caused by impaired mitochondrial metabolism [65]. Using a proteomics-based approach, it was found that concentrations of proteins of the mitochondrial oxidative phosphorylation system, in particular proteins of the ATP synthase complex V such as ATP5C1 and ATP5A1, were significantly lower in dermal fibroblasts from both male and female HGPS patients compared to healthy age- and sex-matched controls [65]. Inversely, proteins involved in glycolysis such as PGK1 and LDH were present at significantly higher levels in HGPS fibroblasts compared to controls [65]. Therefore, it was suggested that dysfunctional mitochondrial respiration results in the activation of glycolytic pathways in HGPS in an attempt to restore the ATP deficiency [65].

Other members of the electron transport chain including cytochrome c, COXI, and β-ATPase were also found to be reduced in HGPS cells [65]. As a result of these relative protein deficiencies, ATP synthesis was decreased by 50% in progeroid mitochondria. These results were confirmed in fibroblasts, skeletal muscle, and heart tissue from progeroid *Lmna^G609G/G609G^* mice which also showed significantly reduced mitochondrial respiratory capacity and COX activity [65]. Interestingly, mitochondrial function was intact in brain tissue of *Lmna^G609G/G609G^* mice—with brain tissue having only negligible levels of progerin—indicating that mitochondrial dysfunction is strictly related to the presence of mutant progerin, contrary to, for example, PXE, where impaired mitochondrial respiration is also present in non-ABCC6 expressing cells such as dermal fibroblasts [35,65].

Finally, the authors showed that treating murine progeroid fibroblasts with FTI-277, a farnesyltransferase inhibitor reducing the levels of toxic farnesylated progerin, significantly increased ATP synthesis and COX levels compared to untreated cells [65]. Similar results were obtained in pravastatin- and zoledronate-treated cells [65]. Interestingly, statin and bisphosphonate treatments have also proven effective in ameliorating EC in *Abcc6^−/−^* mice, though their effects on mitochondrial function and progerin levels (which also accumulates in non-laminopathy disorders and even in physiological ageing) have not been investigated yet in PXE [66,67,68].

Complementary to the findings on mitochondrial dysfunction, increased oxidative stress has also been found in HGPS patient-derived fibroblasts. In an earlier study by Viteri et al., higher levels of ROS as well as oxidized protein and MnSOD content were detected in HGPS cells compared to controls [69]. Together with a decreased proteasome activity, this results in a progressive accumulation of oxidized proteins causing severe cellular dysfunction in HGPS cells [69].

Using a high-throughput screening platform, Kang et al. further unraveled the molecular mechanisms underlying increased ROS production in HGPS [70]. The rho-associated protein kinase (ROCK) inhibitor Y-27632 was found to significantly reduce ROS levels in HGPS fibroblasts by impeding the ROCK-mediated phosphorylation of RAC1B and subsequent interaction between RAC1B and cytochrome c [70]. Finally, the amount of DNA double-strand breaks as well as the frequency of abnormal nuclear morphology—both hallmarks of HGPS—were also significantly attenuated following Y-27632 treatment, making ROCK inhibition a promising therapeutic avenue for HGPS-related premature cellular senescence [70].

Further mechanistic insights into progerin-associated mitochondrial dysfunction and cellular senescence were very recently provided by Maynard et al. [71]. The authors hypothesized that the NAD^+^-dependent sirtuin-1 (SIRT1), peroxisome proliferator-activated receptor-gamma coactivator 1α (PGC1α), and poly (ADP-ribose) polymerase 1 (PARP1) pathways could potentially link the progerin-induced alterations in the nucleus to the impaired mitochondrial respiration as these nuclear pathways are well-known to be involved in regulating mitochondrial function. In their study, Maynard et al. first confirmed that the overall oxygen consumption rate is indeed significantly decreased in murine *Lmna^−/−^* fibroblasts, after which they identified reduced levels of both NAD^+^, SIRT1, and PGC1α in these cells as well as increased levels of acetyl-p53 [71]. Following PARP1 inhibition by means of olaparib treatment, mitochondrial respiration significantly improved [71]. After the induction of oxidative stress by H_2_O_2_, PARylation of proteins increased while the oxygen consumption rate further decreased, suggesting that accumulation of DNA damage-induced PARylation—which excessively consumes NAD^+^—contributes to worsening mitochondrial bioenergetics [71].

It is important to note that decreased SIRT1—a known enhancer of mitochondrial biogenesis and respiration—as well as increased p53 and PAR/PARP1 levels are also present in PXE and contribute to the EC process, as was demonstrated in our previous work [39].

### Conclusion and Outstanding Questions

Similar to PXE, the important role of mitochondrial dysfunction and oxidative stress in the pathophysiology of HGPS has only recently been discovered and acknowledged. Emerging evidence now shows that aberrant lamin A/C protein functioning directly and significantly contributes to impaired mitochondrial respiration and ROS production, mediated via excessive DNA damage response signaling, as is also the case in PXE.

HGPS patients experience extremely accelerated ageing-related signs and symptoms such as insulin resistance, myocardial infarction and stroke, resulting in early mortality and thus presenting a highly unmet medical need. While lonafarnib—a farnesyltransferase inhibitor—is currently FDA-approved for the treatment of HGPS based on a single-arm clinical study [72], additional research into disease-mitigating drugs targeting either progerin toxicity or secondary mitochondrial dysfunction is highly warranted to further extend the lifespan of HGPS patients.

## 5. Mitochondrial Dysfunction and Oxidative Stress in Hereditary Central Nervous System (CNS) Calcification Diseases

While the calcification of CNS structures such as the choroid plexus or pineal gland is frequently encountered in physiological ageing, basal ganglia or subcortical calcification may be found in young individuals as part of an underlying genetic disease (after exclusion of acquired CNS calcification diseases, e.g., congenital infections, hypo/hyperparathyroidism, systemic lupus, erythematosus, etc.) [1,73]. Indeed, CNS calcification is often a hallmark and diagnostic clue of several hereditary conditions including Aicardi–Goutières syndrome (AGS; OMIM #225750), Cockayne syndrome (OMIM #216400) and Leukodystrophy with Cysts and Calcification (LCC; OMIM #614561).

Hereditary CNS calcification diseases present a clinically heterogenous group of neurological conditions with high variability in age of onset, ranging from neonatal to late-adult onset. Cardinal signs and symptoms typically include progressive cognitive, developmental, and motor dysfunction as well as epileptic seizures, hypotonia, and failure-to-thrive [74]. Diagnostically, CNS calcification is best visualized using computed tomography imaging as the hyperdense calcified lesions may be missed on conventional magnetic resonance imaging.

As several CNS calcification diseases are either caused by mutations in genes encoding mitochondrial proteins (termed ‘mitochondriopathies’, e.g., MELAS (OMIM #540000) or Kearns–Sayre syndrome (OMIM #530000)) or are characterized by impaired mitochondrial respiration and oxidative stress (e.g., *NRROS*-associated neurodegeneration (OMIM #618875)), mitochondrial dysfunction is believed to significantly contribute to the pathogenesis of aberrant calcium crystal deposition in the CNS [75].

Recently, pathogenic variants in the *PNPT1* gene have been identified as a potential cause of Aicardi–Goutières syndrome (AGS), a type I interferonopathy characterized by basal ganglia calcification. In their study, Bamborschke et al. described the case of a 14-month-old girl with a clinical AGS phenotype and significantly increased interferon score, indicative of constitutive type I interferon activation [76]. A novel homozygous, likely pathogenic missense variant was then identified in *PNPT1,* which encodes the polyribonucleotide nucleotidyl-transferase 1 protein mainly localized in the mitochondrial intermembrane space [76]. PNPT1 has been implicated in the import of small noncoding nuclear RNA (e.g., 5S rRNA, MRP RNA) into mitochondria, contributing to adequate mitochondrial translation and respiratory chain functioning [77]. Indeed, Vedrenne et al. showed that bi-allelic *PNPT1* mutations result in a significantly decreased import of 5S rRNA into mitochondria in the fibroblasts of an affected patient as well as a decreased mitochondrial translation rate (mainly affecting COX subunits) and reduced activity of respiratory chain complexes III and IV [78]. Conversely, overexpression of PNPT1 rescued the RNA import deficit in patient fibroblasts [78].

Additionally, PNPT1 deficiency has been shown to result in an accumulation of double-stranded mtRNA in mitochondria and its subsequent release into the cytoplasm, where it is recognized by the pattern recognition receptor MDA5, triggering the activation of antiviral type I interferon pathways [79,80]. CNS calcification is then believed to occur due to interferon-mediated neuroinflammation and vascular damage.

Bi-allelic loss-of-function variants in the *NRROS* (Negative Regulator of Reactive Oxygen Species) gene cause an early-onset and severe neurodegenerative disorder characterized by punctate calcifications in the subcortical and periventricular white matter [81,82]. NRROS negatively regulates the NADPH oxidase NOX2, thus preventing excessive ROS production [83]. Additionally, NRROS has also been shown to activate latent TGF-β1, thereby controlling microglial activation and neuroinflammatory responses [84].

Postmortem pathological examination of the brain tissue of a 3-year-old female harboring *NRROS* mutations revealed severely reduced white matter with diffuse gliosis, dystrophic calcifications, and widespread infiltration of foamy macrophages [85].Globular mitochondria with concentrically arranged cristae were observed at the ultrastructural level [85]. Taken together, it is hypothesized that *NRROS* mutations result in increased ROS generation due to (I) deficient inhibition of NOX2 and (II) failure to release active TGF-β1, thus causing uncontrolled activation of microglia and inflammation-related ROS secretion from mitochondria [85].

*KARS*-related progressive leukoencephalopathy (OMIM #619147) is a highly heterogenous clinical entity characterized by typical CNS calcifications in the brainstem and spinal cord [86]. *KARS* encodes the lysyl-tRNA synthetase which catalyzes the aminoacylation of tRNA-Lys in mitochondria and in the cytosol. On CT imaging, a sometimes spectacular CNS calcification phenotype is observed, with calcium crystal deposits in the pons, thalamic nuclei, cerebellum, and periventricular white matter as well as ‘track-like’ calcifications in the entire spinal cord (anterior horns) [86]. Biochemically, multi-complex mitochondrial respiratory chain defects have been identified in patient fibroblasts and muscle biopsies, often accompanied by elevated lactate levels (typically in patients with cardiac involvement) [86].

Remarkably, while KARS is both present in mitochondria and the cytoplasm, pathogenic variants in this gene only resulted in impaired mitochondrial translation while cytosolic translation was unaltered [87]. Rescue experiments showed that reintroducing mitochondrial KARS, but not the cytosolic isoform, normalized mitochondrial translation [87]. Finally, protein levels of oxidative phosphorylation components such as complex I, III, and IV were significantly reduced in *KARS*-mutant fibroblasts compared to controls, confirming the role of KARS in mitochondrial respiratory chain biogenesis [87].

Primrose syndrome (OMIM #259050) is an autosomal dominant neurodevelopmental disorder presenting a peculiar EC phenotype characterized by calcification of the external ear and, to a lesser extent, of the brain parenchyma [88]. Calcification of the ears is largely age-dependent, being present in only a minority of affected children, though it reaches 100% penetrance in adults [88]. Mutations in the *ZBTB20* gene, a transcriptional repressor of the zinc finger family, underlie Primrose syndrome [89]. Both metabolic disturbances (abnormal acylcarnitine and urine organic acid profiles including increased excretion of dicarboxylic acids, ethylmalonic, and glutaric acids) and clinical signs (lipodystrophy and impaired glucose tolerance) are highly suggestive of mitochondrial dysfunction, which has also been observed in *Zbtb20* knock-out mice [88,90]. However, whether Primrose syndrome is truly characterized by impaired mitochondrial functioning needs to be further investigated in future studies.

Lastly, MELAS (mitochondrial Myopathy, Encephalopathy, Lactic Acidosis and Stroke-like episodes) is a hereditary disorder caused by mutations in either mitochondrial tRNA genes (including *MTTL1*) or mitochondrial complex I genes [75]. Bilateral calcification of the basal ganglia is a hallmark feature of the disease and is encountered in the majority of patients. A recent metabolomics study by Sharma et al. revealed that mitochondrial markers such as α-hydroxybutyrate, β-hydroxy acylcarnitines, and β-hydroxy fatty acids were significantly altered in plasma from MELAS patients compared to controls and, perhaps more importantly, correlated strongly with MELAS disease severity [91]. Interestingly, plasma metabolic profiles from MELAS patients were mainly characterized by elevated NADH-reductive stress (increased NADH/NAD^+^ ratio) [91]. Furthermore, distinct plasma metabolic abnormalities may be directly associated to specific MELAS phenotypes. For example, increased α-hydroxybutyrate concentrations are well-known to be involved in the pathogenesis of diabetes mellitus [91].

Several mitochondrial abnormalities including reduced membrane potential, decreased basal respiration, increased MnSOD levels, and high oxidative stress have been reported in MELAS fibroblasts and can be attenuated following treatment with rapamycin, an inhibitor of the mTOR protein kinase [92]. Further research is needed to evaluate the therapeutic potential of mTOR inhibitors in alleviating clinical symptoms in MELAS, a currently intractable and fatal disease.

### Conclusion and Outstanding Questions

As illustrated above, hereditary CNS calcification disorders comprise a broad group of disease entities characterized by significant clinical, biochemical, and genetic variability. For the majority of these orphan diseases, the currently available data are limited and mostly comprise clinical case reports with only sporadic experimental data evaluating mitochondrial function. Additionally, significant gaps persist in our knowledge regarding the precise molecular link between ‘neuroinflammation,’ which is present in many of these disorders, and the EC process itself.

Finally, as targeting soft tissue calcification in ‘easily accessible’ peripheral organs such as the skin or blood vessels has already shown to be complicated, pharmacological interference with calcification processes in the highly-restricted CNS—which is additionally protected by the lipophilic blood-brain barrier—may well be the most difficult challenge lying ahead.

## 6. Conclusions

**Figure 1:**
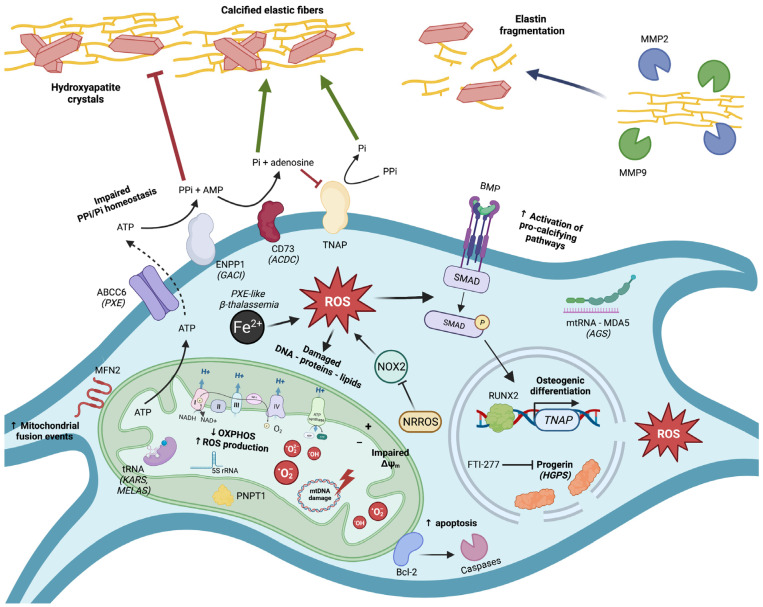
Graphical representation of key molecular pathways involved in mitochondrial dysfunction, oxidative stress, and subsequent ectopic calcification. ABCC6 = ATP-binding cassette subfamily C member 6; ACDC = arterial calcification due to CD73 deficiency; AGS = Aicardi–Goutières syndrome; AMP = adenosine monophosphate; ATP = adenosine triphosphate; Bcl-2 = B-cell lymphoma 2; BMP = bone morphogenetic protein; CD73 = cluster of differentiation 73; ENPP1 = ectonucleotide pyrophosphatase/phosphodiesterase 1; FTI-277 = farnesyl transferase inhibitor-277; GACI = generalized arterial calcification of infancy; HGPS = Hutchinson–Gilford progeria syndrome; KARS = lysyl-tRNA synthetase 1; MDA5 = melanoma differentiation-associated protein 5; MELAS = mitochondrial encephalopathy, lactic acidosis and stroke-like episodes; MFN2 = mitofusin 2; MMP = matrix metalloproteinase; mtRNA = mitochondrial ribonucleic acid; NAD = nicotinamide adenine dinucleotide; NOX2 = NADPH oxidase 2; NRROS = negative regulator of ROS; OXPHOS = oxidative phosphorylation; Pi = inorganic phosphate; PNPT1 = polyribonucleotide nucleotidyltransferase 1; PPi = inorganic pyrophosphate; PXE = pseudoxanthoma elasticum; ROS = reactive oxygen species; rRNA = ribosomal ribonucleic acid; RUNX2 = runt-related transcription factor 2; SMAD = suppressor of mothers against decapentaplegic; TNAP = tissue-nonspecific alkaline phosphatase; tRNA = transfer ribonucleic acid.

Mitochondrial dysfunction and oxidative stress are frequently encountered in hereditary EC diseases and have direct and substantial links to key pathways involved in soft tissue calcification such as PPi homeostasis, calcium metabolism, and apoptosis (Figure 1). Significant insights into mitochondrial (mal)functioning and its far-reaching local and systemic consequences have been obtained by studying these rare genetic disorders, and are applicable to more common diseases, such as CKD-associated vascular calcification, which impose a large disease burden at the population level.

By studying and comparing both the mitochondriopathies, in which the link to mitochondrial dysfunction is typically clear, and EC diseases such as PXE, in which mitochondrial impairment was initially considered to be only a secondary and negligible epiphenomenon, researchers may be able to carefully dissect the different molecular signatures resulting from either mitochondrial dysfunction as a cause of disease or from mitochondrial dysfunction as a consequence of disease. In the case of PXE, this question is highly debated and its future answers will determine whether clinical trials with therapeutic compounds targeting oxidative stress and mitochondrial impairment will ever be pursued in this patient population.

Additionally, fundamental research into mitochondrial dysfunction in other Mendelian EC diseases closely related to PXE, such as PXE-like syndrome with multiple coagulation factor deficiency (OMIM #610842), GACI, and arterial calcification due to deficiency of CD73 (ACDC; OMIM #211800) is highly warranted. Interestingly, the study of Mori et al. has hinted towards a direct link between ENPP1 and mitochondrial dysfunction as it was shown that protein carbamylation exacerbated VSMC calcification by increasing mitochondria-derived oxidative stress, which in turn downregulated ENPP1 expression [93]. As a substantial clinical and molecular overlap exists between PXE and these disorders, it is thus likely that disturbed mitochondrial bioenergetics also contributes to their pathogenesis.

Finally, it is noteworthy that other sources of ROS including endothelial nitric oxide synthase (eNOS) uncoupling may also contribute to the oxidative stress burden in hereditary EC diseases, warranting further research.

## Figures and Tables

**Table 1:** Overview of hereditary ectopic calcification diseases with known mitochondrial dysfunction and/or oxidative stress.

Disease	Causal Gene(s)	Clinical EC Phenotype	Mitochondrial Dysfunction/Oxidative Stress
Pseudoxanthoma elasticum (PXE)	*ABCC6* (or *ENPP1*)	EC in reticular dermis (skin), Bruch’s membrane (eye), tunica media (artery) and papilla (kidney)	Swollen mitochondria, ↓ cristae, ↓ OCR, ↑ fusion events, ↑ ∆ψ_m_, ↑ ROS, ↑ MDA/AOPP, ↓ TAS, ↓ Bcl-2, ↑ protein oxidation
β-thalassemia/sickle cell anemia	*HBB*	PXE-like EC in skin, eyes (angioid streaks), and arteries	↑ LOOH/AOPP/MDA, ↑ SOD and GPX↓ NF-E2—ABCC6 association, ↑ ROS (due to ↑ Hb degradation + iron overload)
Hutchinson–Gilford progeria syndrome (HGPS)	*LMNA*	Cardiovascular calcification (tunica media)	↓ ATP production, ↓ ATP synthase (complex V), ↓ cytochrome c, ↑ ROS, ↑ protein oxidation, ↓ OCR, ↑ SOD
*PNPT1*-associated Aicardi–Goutières syndrome (AGS)	*PNPT1*	Basal ganglia calcification	↓ Mitochondrial import of 5S rRNA, ↓ mitochondrial translation, ↓ complex III/IV activity
*NRROS*-associated neurodegeneration	*NRROS*	Puntacte calcifications in subcortical and periventricular white matter	Globular mitochondria with concentrically arranged cristae, hypothesized ↑ ROS due to ↑ NOX2
*KARS*-related progressive leukoencephalopathy	*KARS*	Brain (pons, thalamus, cerebellum, and white matter) and spinal cord calcifications	↓ Mitochondrial translation, ↓ complex I/II/IV protein levels
Primrose syndrome	*ZBTB20*	Calcification of the external ear and brain parenchyma	Not established but abnormal acylcarnitine and urine organic acid profiles (↑ excretion of dicarboxylic acids, ethylmalonic and glutaric acids)
Mitochondrial myopathy, encephalopathy, lactic acidosis, and stroke-like episodes (MELAS)	Mitochondrial tRNA *(MTTL1)* or complex I genes	Bilateral basal ganglia calcification	Altered mitochondrial metabolome (acylcarnitine, β-OH fatty acids), ↓ ∆ψ_m_, ↓ basal respiration, ↑ SOD, ↑ ROS

## Data Availability

Not applicable.
